# Improving Species Distribution Modelling of freshwater invasive species for management applications

**DOI:** 10.1371/journal.pone.0217896

**Published:** 2019-06-17

**Authors:** Marta Rodríguez-Rey, Sofia Consuegra, Luca Börger, Carlos Garcia de Leaniz

**Affiliations:** Department of Biosciences, Swansea University, Swansea, United Kingdom; Universidade Federal de Goias, BRAZIL

## Abstract

Freshwater ecosystems rank among the most endangered ecosystems in the world and are under increasing threat from aquatic invasive species (AIS). Understanding the range expansion of AIS is key for mitigating their impacts. Most approaches rely on Species Distribution Models (SDMs) to predict the expansion of AIS, using mainly environmental variables, yet ignore the role of human activities in favouring the introduction and range expansion of AIS. In this study, we use five SDM algorithms (independently and in ensemble) and two accuracy measures (TSS, AUC), combined with a null modelling approach, to assess the predictive performance of the models and to quantify which predictors (environmental and anthropogenic from the native and introduced regions) best explain the distribution of nine freshwater invasive species (including fish, arthropods, molluscs, amphibians and reptiles) in a large island (Great Britain), and which species characteristics affect model performance. Our results show that the distribution of invasive species is difficult to predict by SDMs, even in cases when TSS and AUC model accuracy values are high. Our study strongly advocates the use of null models for testing SDMs performance and the inclusion of information from the native area and a variety of both human-related and environmental predictors for a more accurate modelling of the range expansion of AIS. Otherwise, models that only include climatic variables, or rely only on standard accuracy measures or a single algorithm, might result in mismanagement of AIS.

## Introduction

Developing a scientific basis for monitoring and managing invasive species and implementing measures to manage pathways to prevent introductions is one of the CBD Aichi Targets for 2020 [1]. Freshwater invasions are of special concern, as freshwater ecosystems are among the most diverse and endangered ecosystems in the world [2], harbouring more than a quarter of all freshwater fauna threatened or recently extinct [3], in part due to the impact of non-native freshwater species on native biodiversity [4]. Despite an increase in the number of studies focusing on freshwater invasions in recent years [5], the main drivers of the introduction and spread of aquatic invasive species (AIS) are still unknown [6].

Species Distribution Models (SDMs) have widely been used as a management tool for AIS [7]. These correlative techniques allow to model the distribution of species and map the spatial suitability of areas based on the identification of statistical associations between species’ occurrence and predictor variables [8]. SDM outputs can be used for predicting changes in species’ distributions under environmental change and devise conservation and management strategies [9]. Recent studies using SDMs have generated estimates of habitat suitability for AIS mainly using environmental variables [7], based on the assumption of a natural colonisation pattern, whereby species increase their range in areas with favourable environmental conditions. This approach, using only climatic variables, has allowed the development of models for forecasting invasive species’ distributions under future scenarios of climate change [10]. However, human-mediated range-shifts, although less predictable, may play a larger role than climate change in driving the expansion of AIS [11].

Human-related factors play indeed a fundamental role in the introduction and dispersal of invasive species [12, 13], and accordingly, consideration of human mediated dispersion appears essential for improving the explanatory and predictive accuracy of models [14]. Existing SDMs studies have incorporated variables such as human population density or presence of roads [15] to account for the effect of human-mediated dispersal, but more detailed human-related variables are required to account for propagule pressure (e.g. aquaculture, horticulture, shipping frequency) [16].

Here, we assessed the relative ability of human-mediated and environmental predictors to model the invasion of nine AIS belonging to five broad taxa (molluscs, arthropods, fish, amphibians, and reptiles) in Great Britain, as a case study. We included environmental variables from both the native and invaded ranges of the species (to predict their potential eco-physiological range) and human-related variables (to predict their human-induced geographical range), and tested model performance in relation to: (*i*) type of predictors (environmental in the native and invaded region, environmental only in the invaded region or environmental and anthropic in the invaded region) and (*ii*) characteristics of the species’ spatial records and their invasion (e.g., time since first introduction, economic interest, distance between the southernmost and northernmost records).

**Fig 1:**
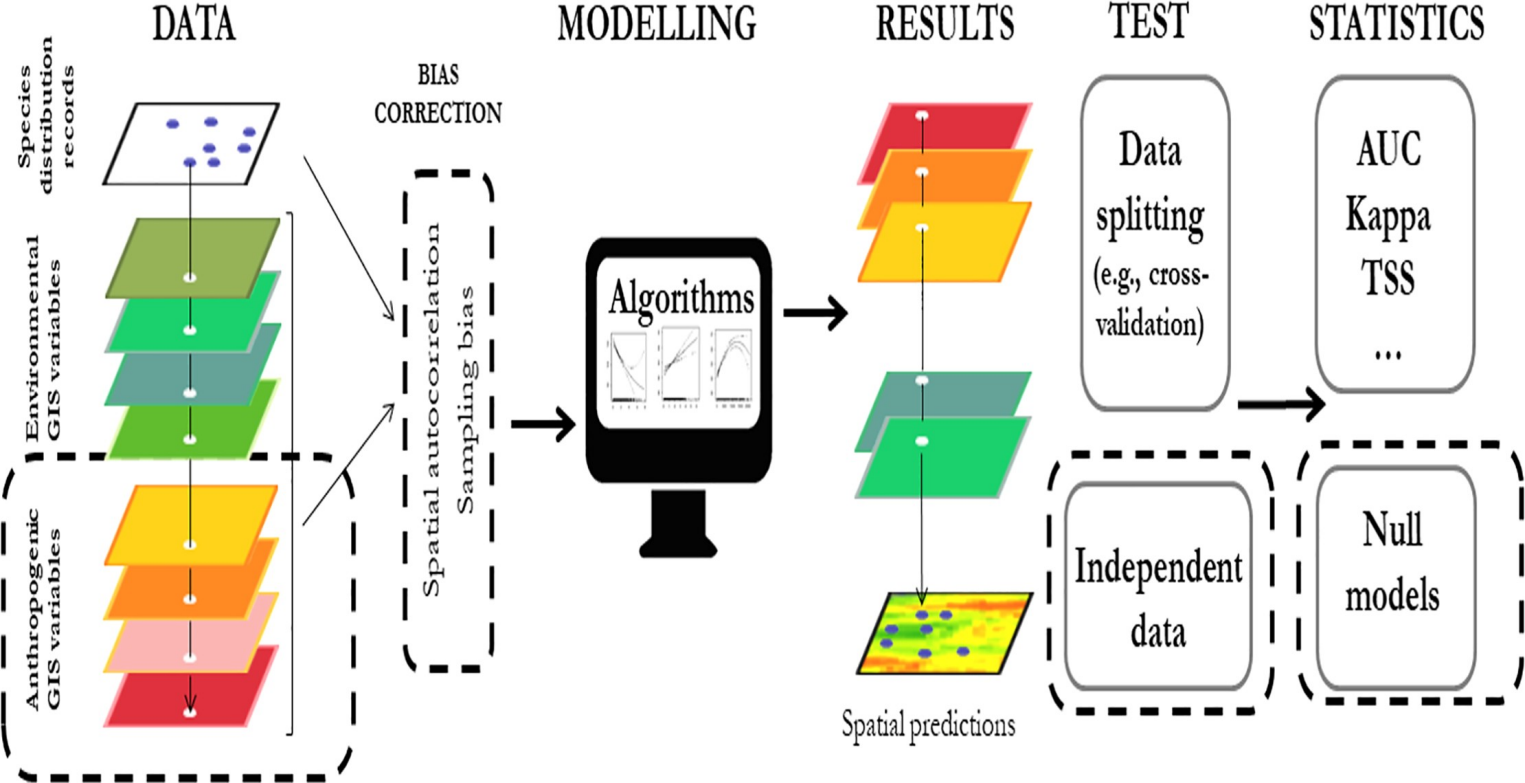
Diagram of the Species Distribution Modelling procedure. Dashed boxes mark the parts of the approach that have been improved in our study as compared to the common procedure.

To test the predictive ability of the different models, our approach differs from similar studies on invasive species in that it includes a large range of anthropic variables [17], control of most important biases [18] temporally independent evaluation [17, 19] and a robust approach based on TSS and AUC statistics combined with comparisons to null models [20] (Fig 1).

## Materials and methods

### Study area and species

Islands provide good opportunities for studying invasion processes due to their isolation–here we used Great Britain. We divided the study area into 5x5 Km^2^ grid cells but excluded those with less than 70% of the grid area (typically, coastal ones), giving a total of 8,735 valid cells. We used this grid resolution to avoid streams from different catchments being present in the same grid and to retain as many presence/absence records as possible. Grid cells have been previously used as reference area to study the distribution of freshwater species in broad areas when using river fragment as reference is arbitrary and computationally tedious [13, 21, 22]. We modelled the distribution of nine species from five different taxa (fish, arthropods, molluscs, amphibians and reptiles): the wels catfish (*Silurus glanis*), pumpkinseed (*Lepomis gibbosus*), zander (*Sander lucioperca*) and sunbleak (*Leucaspius delineates*) amongst the fish; signal crayfish (*Pacifastacus leniuscus*), killer shrimp (*Dikerogammarus villosus*) amongst the arthropods; the zebra mussel (*Dreissena polymorpha*) among the molluscs; the marsh frog (*Pelophylax ridibundus*) amongst the amphibians, and the red-eared slider (*Trachemys scripta*) among the reptiles. Species occurrence records in the invaded region were obtained from the NBN Gateway database (http://www.nbn.org.uk/), which is the most complete source of non-native species distribution data in Great Britain [23] and species occurrence in the native area were obtained from the Global Biodiversity Information Facility (GBIF, http://gbif.org).

To account for sampling bias [24] we compared the distribution of the nine invasive study species with the distribution of similar native species for each taxon. This comparison accounted for the number of presence of native species in the absence of invasive species. The purpose of this analysis was to make sure that grid cells included in our study had been sampled for similar species, providing greater confidence on the absence of AIS [25, 26]. We used data from the NBN database to compare the distribution of invasive fish in Great Britain with those of brown trout (*Salmo trutta*), Atlantic salmon (*Salmo salar*), spined loach (*Cobitis taenia*), European bullhead (*Cottus gobio*) and Allis shad (*Alosa alosa*); we used the distribution of the common frog (*Rana temporaria*) and the common toad (*Bufo bufo*) for amphibians, and the distribution of 21 species of *Gammarus* was used as a control for the killer shrimp. We could not find comparable data for the distribution of the red-eared slider, the zebra mussel and the signal crayfish since there are no native sliders in Great Britain and the native freshwater pearl mussel (*Margaritifera margaritifera*) and the white-clawed crayfish (*Austropotamobius pallipes*) are critically endangered, or endangered, respectively, and their current distributions would not be representative.

We used data from GBIF to compare the distribution in the native area of the pumpkinseed with the largemouth bass (*Micropterus salmoides*) which is a well sampled species due to its interest as a game species, the signal crayfish with the pilose crayfish (*Pacifastacus gambelii*) the only native crayfish sharing distribution range [27], and the red eared slider with the southern painted turtle (*Chrysemys picta*) and river cooter (*Pseudemys concinna*). Wels catfish, sander and sunbleak were compared to barbel (*Barbus barbus*), northern pike (*Esox lucius*) and gudgeon (*Gobio gobio*).

Pseudo-absences and background data corresponded to those areas without invasive species. We randomly selected pseudo-absences after checking the sampling effort, instead of incorporating the sampling effort to the model because most invasive species had more than 70% of their area sampled; justified by the presence of at least another related species or lack of sampling bias information [25, 26]. We also used this approach to avoid bias towards places with a high number of occurrence records, as many invasive species might be introduced in remote places where sampling is less common due to the accessibility [24].

As the spatial domain of the study area needs to be constrained in order to retain only meaningful ecological variables [28], we excluded those cells that were 200 kilometres beyond the northernmost presence for each species in the invaded area, and used a convex hull of occurrences in the native area, to obtain a more representative environmental domain [29].

### Modelling scenarios and predictor variables

Three different scenarios were used based on the inclusion of different types of predictors: (i) environmental variables only in the invaded area (INVADED), (ii) environmental variables in both the native and the invaded area (NATIVE) and (iii) environmental and anthropic variables in the invaded area (MIXED). Anthropogenic variables cannot be combined with the native range since they usually have the contrary effect than in the invaded area since human activities use to negatively impact native species distribution ranges [30]. We used 19 bioclimatic variables, as well as altitude and slope as topographic variables, and land use as a proxy for the fundamental niche [9, 31]. We extracted the mean values of the grid cells of all the variables (Table 1) in each grid cell using the zonal statistic tool in QGIS [32]. Using the hydrography map, we estimated land use predictors within a 50 m buffer strip from each river bank.

**Table 1:** Predictor variables used to generate the Species Distribution Models. Variables in bold had VIF scores smaller than 10 [33] and were included in the Species Distribution Models.

Predictor	Variable	Source	Description
	**Distance to the first record**	**https://data.nbn.org.uk/ and http://www.nonnativespecies.org/factsheet/**	Euclidean distance from the first record reported in the database and in accordance with each species factsheet.
**ENVIRONMENTAL**	**Slope**	**http://www.sharegeo.ac.uk/handle/10672/7**	Mean slope in each grid obtained from a Digital Elevation Model
	**Altitude**	**http://www.sharegeo.ac.uk/handle/10672/5**	Mean slope in each grid obtained from a Digital Elevation Model
	Climatic Bio1	http://www.worldclim.org/bioclim	Annual Mean Temperature
	Climatic Bio 2	http://www.worldclim.org/bioclim	Mean Diurnal Range (Mean of monthly (max temp—min temp))
	**Climatic Bio 3**	**http://www.worldclim.org/bioclim**	Isothermality (BIO2/BIO7) (* 100)
	**Climatic Bio 4**	**http://www.worldclim.org/bioclim**	Temperature Seasonality (standard deviation *100)
	Climatic Bio 5	http://www.worldclim.org/bioclim	Max Temperature of Warmest Month
	**Climatic Bio 6**	**http://www.worldclim.org/bioclim**	Min Temperature of Coldest Month
	Climatic Bio 7	http://www.worldclim.org/bioclim	Temperature Annual Range (BIO5-BIO6)
	**Climatic Bio 8**	**http://www.worldclim.org/bioclim**	Mean Temperature of Wettest Quarter
	**Climatic Bio 9**	**http://www.worldclim.org/bioclim**	Mean Temperature of Driest Quarter
	Climatic Bio 10	http://www.worldclim.org/bioclim	Mean Temperature of Warmest Quarter
	Climatic Bio 11	http://www.worldclim.org/bioclim	Mean Temperature of Coldest Quarter
	Climatic Bio 12	http://www.worldclim.org/bioclim	Annual Precipitation
	Climatic Bio 13	http://www.worldclim.org/bioclim	Precipitation of Wettest Month
	Climatic Bio 14	http://www.worldclim.org/bioclim	Precipitation of Driest Month
	**Climatic Bio 15**	**http://www.worldclim.org/bioclim**	Precipitation Seasonality (Coefficient of Variation)
	Climatic Bio 16	http://www.worldclim.org/bioclim	Precipitation of Wettest Quarter
	Climatic Bio 17	http://www.worldclim.org/bioclim	Precipitation of Driest Quarter
	**Climatic Bio 18**	**http://www.worldclim.org/bioclim**	Precipitation of Warmest Quarter
	Climatic Bio 19	http://www.worldclim.org/bioclim	Precipitation of Coldest Quarter
	**Land Uses: Grasslands in the riverside**	**CORINE Land Cover** **http://land.copernicus.eu/pan-european and North American Land Cover Monitoring System (NALCMS) http://www.cec.org/tools-and-resources/map-files/land-cover-2010-landsat-30m**	Percentage of grassland and cropland in a 100 m buffer along the river
	**Land Uses: Natural forest in the riverside**		Percentage of preserved forest in a 100 m buffer along the river
**ANTHROPIC**	**Lakes/ Reservoir**	**https://www.sharegeo.ac.uk/**	Percentage of lakes and/or reservoirs
	**Distance to cities > 100K population**	**Own creation based on data from Office of National Statistics**	Euclidean distance to human settlements with more than 100000 inhabitants based on 2011 census
	**Population density**	**Diva-GIS http://www.diva-gis.org/Data**	Population density
	Distance to pet stores	Own creation	Average Euclidean distance to the closest pet stores
	**Distance to garden centers**	**Own creation**	Average Euclidean distance to the closest garden centres
	**Distance to farms**	**Own creation**	Average Euclidean distance to the closest aquaculture facility, farm or hatchery
	**Road density**	**https://www.ordnancesurvey.co.uk/**	Kilometres of road
	**Distance to Boat Launch**	**http://www.boatlaunch.co.uk/#/map**	Euclidean distance to the closest freshwater boat launch
	**Distance to Ports**	**https://www.sharegeo.ac.uk/**	Euclidean distance to the closest port
	**Canal density**	**https://www.sharegeo.ac.uk/**	Meters of river channels

In order to model human-mediated geographical range expansion (i.e. promoted by anthropically mediated spread and/or propagule pressure), we included the distance to the closest town and city (with more than 100,000 habitants), and population density as an indicator of human presence and pressure. As an indicator of human accessibility, we used road density, distance to the nearest port, and distance to the nearest boat launch ramp. We also included density of reservoirs, lakes, and canals as an indicator of building infrastructures and places that may facilitate the spread and/or arrival of AIS through human activities like angling, canoeing or boating [34]. Aquaculture facilities, garden centres and pet stores have been reported to be sources of introduction and dispersal of AIS [35]. For that reason, we included the distance to these three types of facilities to account for potential sources of introduction and propagule pressure. All proximity variables (e.g., distance to boat launch ramp or to pet stores) were calculated by creating a distance map in QGIS and then extracting the mean values for each grid cell. All models using the invaded area included the Euclidean distance to the first record of introduction to account for spatially-correlated patterns of dispersal, as this approach has previously been shown to perform better than more complex SDMs models [36, 37]. We accounted for collinearity by calculating the Variance Inflation Factor [VIF [38], based on the R-squared value of the regression of one variable against all other variables and defined as VIF__j_ = 1/(1-R_j^2),. Twelve bioclimatic variables were excluded due to multicollinearity (VIF > 10; see Table 1), retaining 23 variables for modelling [39]. For each scenario and species, the distribution was analysed using five independent SDMs algorithms: Generalised Additive Models [GAM [40]] using the *mgcv* package in R [41] and MaxEnt [42] using the *dismo* package [43], Boosted Regression Trees [BRT [44]], Generalized Linear Models [GLM [45]] and Random Forest models [RF [46]]; and an ensemble model including all these algorithms. For this, we used *biomod2* package in R [47] and the ensemble was calculated by averaging model predictions weighted by ROC and TSS. We included single algorithms and ensemble to minimise the uncertainty of the techniques selection and outputs[48].

### Training and testing data

We divided the presence data on the invaded region into training and testing data sets for each species based on the date the species were first reported in a given locality. This approach is more robust than randomly selecting training and testing presence data [19, 49, 50]. It also allows the evaluation of each model using a temporally independent validation to recreate the invasion process followed by the species and investigate if the patterns detected by the models at time 1 are similar at time 2[37, 51, 52]. We selected the 70% oldest records for training and the remaining 30% of records (i.e. most recent) for testing. For the NATIVE models, we included all the presence records available in the native region in addition to the 70% of records of the invaded region to account for whole range of conditions where the species are able to persist [53]. For training pseudo-absences, we randomly chose the same number of grid cells as the presence for each species to minimize the potential effect of prevalence. Testing pseudo-absences were selected based on the correction for sorting bias (see below).

Spatial sorting bias may pose a problem due to spatial autocorrelation and tends to generate inflated model results [54]. We removed this bias by pairwise distance sampling implemented in the *dismo* package in R [43].

Model performance was assessed using True Skills Statistic [TSS [55]] and the Area Under the Curve [AUC, [56]] measures of accuracy. Although the idea that SDMs perform better than random when AUC is > 0.5 [57] and/or TSS is > 0 [55] is widespread, this makes the implicit assumption that there is no spatial sorting bias in the evaluation data. For this reason, we built null models to assess whether the performance of the SDMs for each species, algorithms/ensemble and scenario was better than expected by chance. This is a suggested approach when using pseudo-absences that allows for significance testing of SDMs [20]. Null model can be based on randomizations of ecological data or random sampling from a distribution [58]. In our study, the null models (i.e. those expected by chance alone) were created using the same training and testing distribution data used for the real models but with randomly reshuffled predictors. For each species, algorithms/ensemble and scenario we obtained 1000 null models, each one with different rearrangement of the predictors [20, 59] obtained by permutation using base R [60]. We then run 1,000 permutations for each model, and as for the real models, we then calculated performance statistics as above.

In order to extract the distribution of performance statistics (i.e. TSS and AUC) for the null models (thereafter TSSnull and AUCnull), we assessed the upper limit of the 95 confidence interval by calculating the 97.5 percentile in the distribution of the 1,000 performance statistics generated for each null model. Then, we calculated the differences in the accuracy measures between the null model and the real model by subtracting the real model statistic value by the null model statistic value (thereafter ‘effect size’, AUCes and TSSes). Hence, positive values indicate that the model performed better than null models, whereas negative values indicate that the model performed worse than null models (i.e. no better than random).

We obtained the best model for the nine species for each of the five different algorithms (GAM, MaxEnt, BRT, RF and GLM) and the ensemble model, and the two values of SDM performance (i.e., TSSes and AUCes) to determine which overall scenario (i.e. Native, Invaded or Mixed) predicted the species distribution best. To analyse the importance of scenario, algorithm and, the characteristics of the species’ records and species invasion (see Table 2) in the performance, we used the values of all the models better than null and employed linear mixed models (LMM) for each performance metrics (AUCes and TSSes) considering species as random factor.

**Table 2:** Characteristics of the species’ spatial records and their invasion used as predictors to model the overall performance ability of the freshwater invasive Species Distribution Models.

**Species**	**Time since Introduction (yrs.)**	**Economic interest**	**No. presences**	**Distance between N-S occurrences (km.)**
**Zebra mussel**	191	No	376	398
**Red-eared slider**	60	Yes	87	406
**Marsh frog**	133	No	66	230
**Pumpkinseed**	97	No	20	486
**Zander**	138	Yes	115	172
**Killer shrimp**	6	No	8	90
**Signal crayfish**	40	Yes	544	506
**Sunbleak**	21	No	18	182
**Wels catfish**	152	Yes	94	250

Regarding the characteristics of the species, we considered that the ‘time since introduction’, the number of localities occupied by the species (i.e., ‘number of records’) and the ‘distance between the northernmost and southernmost occurrences’ were indicators of the available time for adaptation or species ability to cope with new conditions, potentially affecting model performance. Likewise, proximity between native and invaded region (i.e., ‘native region’) could indicate similarity of conditions whereas ‘economic interest’ might favour the species to be present in particular localities (e.g., recreational areas) which might be easy to predict. Species characteristics were extracted from the factsheet published by GB Non-native Species Information Portal (www.nonnativespecies.org/factsheet/) and the spatial records characteristics were obtained from the species distributions using QGIS. We assessed LMM assumptions by checking residual plots, normality of residuals, and plots of scaled residuals versus fitted values. No significant deviations from linearity or normality were found nor obvious outliers. All analyses were conducted in R 3.3.1 [61].

## Results

Between 71% and 72% of the grid cells with watercourses in Great Britain included at least one record of a native species of the amphibian and fish groups, respectively. In the native area of the different species between a 69% and a 95% of the grids were sampled for at least a related species.

All TSS for the real models (TSS_real_) values but six were higher than 0, and all but 16 AUC_real_ values were higher than 0.5. The average and standard deviation value for the TSS and AUC performance statistics from the real models were 0.25 ± 0.15 and 0.60 ± 0.11 respectively (S1 Table). TSS_null_ values averaged 0.36±0.24 and AUC_null_ values averaged 0.68±0.13. The species with best average results was the red-eared slider with average of TSS and AUC of 0.66 and 0.28, respectively. The species with highest values of performance in the null models was the killer shrimp with average of TSSnull and AUCnull of 0.67 and 0.86, respectively. (S1 Table). The best performance values for the effect size were for the sunbleak according to the AUCes (0.078) and the TSSes (0.25).

The null model approach indicated that models performed better than chance for seven of the nine AIS: signal crayfish, zebra mussel, red-eared slider, zander, wels catfish, marsh frog and sunbleak (S1 Table). Importantly, this approach showed that relying on a given TSS or AUC value is not sufficient to conclude that a model is reliable since the same or similar accuracy measures’ values (AUC and TSS) for the real models were both better and worse than chance according to the null models (S1 Table). For instance, AUC values of 0.58 (Signal crayfish Ensemble Native model) were better than chance according to the null models (0.03 whereas AUC values of 0.7 (Red-eared slider GAM Invaded) or 0.73 (Pumpkinseed GAM Invaded) were worse than null models (S1 Table). Similarly, relying only on a single performance statistic or a single modelling algorithm can mask model uncertainty. Specifically, 26 out of the total of 135 fitted models (19.3%) performed better than expected by chance according to both performance statistics (i.e. TSSes and AUCes), whereas if only one performance statistic is considered (i.e. TSSes or AUCes), the number of valid models increased to 39 (29%) (S1 Table). For example, for the wels catfish, the Mixed and Invaded Scenarios with Maxent performed better than the null model (all AUCes and TSSes = 0.03) whereas using Ensemble or GLM modelling, only the Invaded Scenario was better than chance and only according to one of the effect sizes (TSSes for Ensemble = 0.08 and AUCes for GLM = 0.003), and no GAM and RF models did better than chance. For marsh frog, Mixed MaxEnt, Native GAM and Mixed and Native for RF performed better than expected by chance, whereas no Ensemble or GLM models for this species performed better than the null models. We were unable to obtain results for the BRT null models because the algorithm did not converge. Therefore, this algorithm was only considered as part of the ensemble.

**Fig 2:**
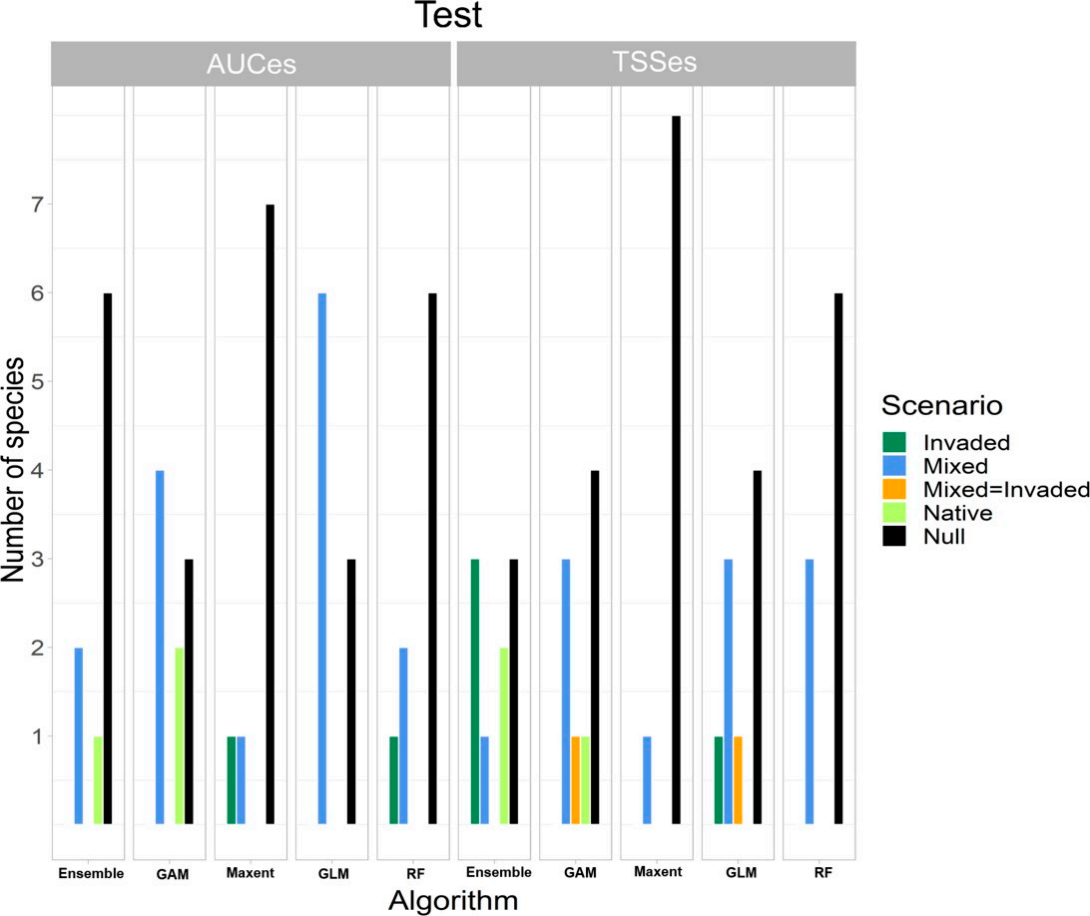
Summary of the best model scenario for the nine invasive species under study according to the three different algorithms/ensemble and the two statistics (i.e. TSS and AUC). ‘Invaded’ scenario included environmental predictors from invaded regions, ‘Native’ included environmental predictors from both native and invaded regions and ‘Mixed’ scenario included environmental and anthropogenic from the invaded region. When two scenario obtained same values another category was created with the ‘ = ‘ symbol to illustrate it.

**Fig 3:**
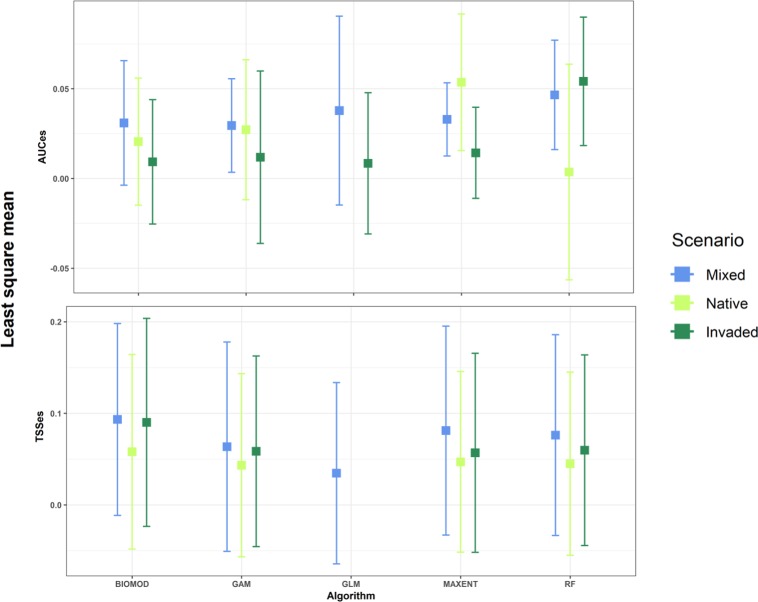
Meanvalues of AUCes and TSSes for the five algorithms with results and the three scenarios. Boxes indicate the least square means based on a linear mixed models considering Scenario and Algorithm. Error bars indicate the 95% confidence interval of the least square means.

Null models performed best in 55% of the cases followed by the Mixed scenario (29%), Native scenario (7%) and Invaded (7%) (Fig 2). According to the Wald test, scenario was a significant predictor of the TSSes (Table 3) and in the LMM the Native Scenario had a negative relationship (estimate = -0.032, sd = 0.01, p<0.01) and Mixed Scenario had a positive relationship (estimate = 0.1, sd = 0.03, p<0.05) with the TSSes. Algorithm and Time since introduction had also a marginally significant relationship with the TSSes (Table 3). None of the considered predictors had an effect on the AUC effect size (Table 3). Least square means and their confidence intervals from the AUCes and TSSes mixed models illustrated the differences in performance for the different algorithms over the different scenarios (Fig 3).

**Table 3:** Results from Wald test of the linear mixed models applied to analyse the relationship between model performance (measured by TSSes and AUCes effect size values) and the type of scenario, algorithm, their interaction and species’ characteristics.

	**AUCes**			**TSSes**		
**Predictor**	Chisq	df	p-value	Chisq	df	p-value
**Scenario**	3.9904	3	0.263	12.2460	3	**0.007**
**Algorithm**	3.5642	4	0.468	9.2427	4	0.055
**Scenario:Algorithm**	7.9929	7	0.333	1.0903	6	0.982
**Distance between northernmost and southernmost records**	0.0313	1	0.860	0.3420	1	0.559
**Number of presences**	0.4068	1	0.524	0.0026	1	0.959
**Economic interest = YES**	0.1582	1	0.691	1.5679	1	0.211
**Time since Introduction**	0.0198	1	0.888	3.6201	1	0.057

## Discussion

We have shown that both environmental (from the native and invaded ranges) and anthropic variables should be included in models that aim to understand and predict the distribution of aquatic invasive species. Our results also highlight the fact that different species may require different sets of predictors and that the inclusion of information about conditions in the species’ native area may be required to model their distribution accurately, making it difficult to generalize across taxa. Therefore, including as much information in the models as possible will help to find the model with the best predictive ability for the species under study, and will permit comparisons between different modelling approaches, as it is not possible to know *a priori* which ones might work best, in agreement with the justification of using ensemble modelling approaches [48].

When the aim is to forecast species’ distributions for management purposes, it has been suggested that the inclusion of anthropogenic variables is essential [16, 62]. For example, human-mediated dispersal may be the only reason for the rapid spread of invasive plants [63], stressing the need to consider a variety of predictors. In practice, many management and conservation plans rely on models that forecast species distributions based only on climatic variables [64], which, according to our study, might result in less effective management of some AIS if resources are invested in addressing the wrong type of drivers. Certainly, models that only use climatic variables are useful to delimit management actions to suitable regions where the species are potentially able to spread without human intervention (i.e., fundamental niche [65]). However, for many species, projecting the distribution of invasive species onto future scenarios of climate change to forecast species expansion will benefit from the integration of anthropogenic variables [16] because human-mediated range-shifts might be more intense than shifts due to climate change [11].

Regarding model performance, our SDMs did not explain the distribution better than chance for two of the nine freshwater invasive species according to the null models. This highlights the difficulties of understanding invasion patterns for some species [66]. Also, the effect sizes (TSSes and AUCes) were low for the better than null models which might be a result of our more robust evaluation of predictive ability, as we accounted for sorting bias and tested the models with a real time independent evaluation.

However, seven species obtained models that performed better than chance even with our robust evaluation procedure, supporting the validity of using SDMs in invasion biology with the right set of predictor variables and algorithms.

AUC and TSS are some of the most commonly used statistics for measuring model accuracy of correlative species distribution models [67]. However, according to our results performance needs to be further assessed, for example, by comparison to null models [59]. AUC validity has been criticised before [68, 69] and although the use of null models for SDMs had been previously recommended [20, 70], this approach has not yet been widely adopted.

None of the characteristics of the species had a significant effect on the performance. However, time since the first introduction was marginally significant for one of the performance metrics (Table 3) so we considered inappropriate to rule out their effects on performance. Previous studies indicated that the characteristics of the species distribution patterns affected the accuracy of SDMs [71]. Time since the first introduction is important for species to adapt and reach suitable areas, making it difficult to discriminate between occupied and unoccupied localities [72]. Newly arrived species typically have small range sizes and, as it has been reported previously [73], modelling species with small number of occurrences or restricted range size requires caution and special protocols particularly in the early stages of invasion, when data limitations make SDMs less accurate [74].

Our study is the first attempt to model freshwater invasions including detailed information from both native and invaded regions and also on anthropic and propagule pressure of different taxa in a relatively isolated system, accounting for bias in the SDMs to avoid overfitting [18]. Our models did not explain the distribution of killer shrimp and pumpkinseed and for other species the predictive performance was low which might be due to the lack of consideration of biological interactions which is one of the factors governing species’ distributions [65]. The use of mechanistic models might further improve our understanding of AIS dispersal [75] by detecting niche shifts during invasion [76]. Given that the number of invasive species continues to increase [77], there is an urgent need to improve predictive management methods since most countries still lack the ability to control invasive species effectively [78].

In conclusion, our study suggests the use of null models to assess model performance will help gain a better understanding of macroecological processes in invasion biology, because relying only on AUC and TSS or a single modelling algorithm is insufficient to obtain reliable models for management. Forecasting invasive species distribution under future scenarios of climate change will be more realistic for a higher number of species if a combination of bioclimatic and anthropogenic variables is considered. Finally, our results also indicate that the distribution of freshwater species with restricted number of records, with higher distances between northernmost and southernmost records and that lack economic interest are particularly difficult to predict, and should therefore be a research priority.

## Supporting information

S1 Table True Skill Statistic (TSS) and Area Under the Curve (AUC) results for real and null models for eleven species, two algorithms and the ensemble, and three scenarios. Effect size corresponds to the difference between the real model and the highest 95CI values of both discrimination statistics.
(DOCX)
