# Supplementary material for: Improving Species Distribution Modelling of freshwater invasive species for management applications
Source: PLoS One. 2019 Jun 17;14(6):e0217896. doi: 10.1371/journal.pone.0217896 (PMC6576753; doi:10.1371/journal.pone.0217896)
Supplement: S1 Table — Effect size corresponds to the difference between the real model and the highest 95CI values of both discrimination statistics. (DOCX) [file pone.0217896.s001.docx]

**Supporting Information**

Improving Species Distribution Modelling of freshwater invasive species for management applications

Marta Rodríguez-Rey*, Sofia Consuegra, Luca Börger & Carlos Garcia de Leaniz

Department of Biosciences, Swansea University, Swansea, UK

Corresponding author: [marta.rodriguez.rey@gmail.com](mailto:marta.rodriguez.rey@gmail.com)

**Table S1**. True Skill Statistic (TSS) and Area Under the Curve (AUC) results for real and null models for eleven species, two algorithms and the ensemble, and three scenarios. Effect size corresponds to the difference between the real model and the highest 95CI values of both discrimination statistics.

| Species | Algorithm | Scenario | TSS_real_ | AUC_real_ | TSS_null_ higher 95CI | AUC_null_ higher 95CI | TSS EffectSize | AUC EffectSize |
| --- | --- | --- | --- | --- | --- | --- | --- | --- |
|  | MaxEnt | Both | 0.267081 | 0.663478 | 0.1863354 | 0.590933 | 0.0725 | 0.0807 |
|  | MaxEnt | Invaded | 0.198758 | 0.613364 | 0.2111801 | 0.6067522 | 0.0066 | -0.0124 |
|  | MaxEnt | Native | 0.151192 | 0.589157 | 0.205864 | 0.605812 | -0.0167 | -0.0547 |
|  | GAM | Both | 0.254658 | 0.665792 | 0.2484472 | 0.6386453 | 0.0271 | 0.0062 |
|  | GAM | Invaded | 0.186335 | 0.601327 | 0.2484472 | 0.640798 | -0.0395 | -0.0621 |
|  | GAM | Native | 0.168063 | 0.596532 | 0.256673 | 0.6254803 | -0.0289 | -0.0886 |
|  | Ensemble | Both | 0.204969 | 0.662359 | 0.111180 | 0.6300008 | 0.0324 | 0.0938 |
| Zebra mussel | Ensemble | Invaded | 0.142857 | 0.609679 | 0.071118 | 0.6047259 | 0.0050 | 0.0717 |
|  | Ensemble | Native | 0.095287 | 0.580256 | 0.100298 | 0.616882 | -0.0366 | -0.0050 |
|  | RF | Both | 0.2298 | 0.647757 | 0.500000 | 0.6878906 | -0.2702 | -0.0401 |
|  | RF | Invaded | 0.1491 | 0.591528 | 0.625000 | 0.828125 | -0.4759 | -0.2366 |
|  | RF | Native | 0.1213 | 0.561952 | 0.6250 | 0.8451 | -0.5038 | -0.2832 |
|  | GLM | Both | 0.2547 | 0.669457 | 0.625000 | 0.8285156 | -0.3703 | -0.1591 |
|  | GLM | Invaded | 0.2236 | 0.610625 | 0.625000 | 0.84375 | -0.4014 | -0.2331 |
|  | GLM | Native | 0.1863 | 0.598623 | 0.625000 | 0.8629 | -0.4387 | -0.2643 |
|  | BRT | Mixed | 0.2485 | 0.6652521 | null | null | null | null |
|  | BRT | Natural | 0.2049689 | 0.6177617 | null | null | null | null |
|  | BRT | Anthropic | 0.2857143 | 0.683307 | null | null | null | null |
|  | MaxEnt | Mixed | 0.315789 | 0.675900 | 0.315790 | 0.645429 | 0.0000 | 0.0305 |
|  | MaxEnt | Invaded | 0.210526 | 0.626731 | 0.342105 | 0.665530 | -0.1316 | -0.0388 |
|  | MaxEnt | Native | 0.289474 | 0.632964 | 0.315790 | 0.612292 | -0.0263 | 0.0207 |
|  | GAM | Mixed | 0.447369 | 0.739612 | 0.394737 | 0.699481 | 0.0526 | 0.0401 |
|  | GAM | Invaded | 0.394737 | 0.699446 | 0.394737 | 0.699481 | 0.0000 | 0.0000 |
|  | GAM | Native | 0.289474 | 0.655125 | 0.342105 | 0.668958 | -0.0526 | -0.0138 |
|  | Ensemble | Mixed | 0.184211 | 0.680748 | 0.236842 | 0.727311 | -0.0526 | -0.0466 |
| Red-eared slider | Ensemble | Invaded | 0.210526 | 0.662050 | 0.236842 | 0.690833 | -0.0263 | -0.0288 |
|  | Ensemble | Native | 0.131579 | 0.619114 | 0.078947 | 0.666776 | 0.0526 | -0.0477 |
|  | RF | Both | 0.368421 | 0.675208 | 0.447368 | 0.729328 | -0.0789 | -0.0541 |
|  | RF | Invaded | 0.210526 | 0.618421 | 0.210526 | 0.618421 | 0.0000 | 0.0000 |
|  | RF | Native | 0.263158 | 0.597645 | 0.342105 | 0.621884 | -0.0789 | -0.0242 |
|  | GLM | Both | 0.421053 | 0.745845 | 0.394737 | 0.714993 | 0.0263 | 0.0309 |
|  | GLM | Invaded | 0.342105 | 0.723684 | 0.421053 | 0.722403 | -0.0789 | 0.0013 |
|  | GLM | Native | 0.184211 | 0.545706 | 0.184211 | 0.545706 | 0.0000 | 0.0000 |
|  | BRT | Mixed | 0.3947368 | 0.6918283 | null | null | null | null |
|  | BRT | Natural | 0.2631579 | 0.6412742 | null | null | null | null |
|  | BRT | Anthropic | 0.2894737 | 0.6585873 | null | null | null | null |
|  | MaxEnt | Mixed | 0.392857 | 0.660714 | 0.357143 | 0.656952 | 0.0357 | 0.0038 |
|  | MaxEnt | Invaded | 0.214286 | 0.609694 | 0.357143 | 0.662022 | -0.1429 | -0.0523 |
|  | MaxEnt | Native | 0.289474 | 0.632964 | 0.340179 | 0.645855 | -0.0507 | -0.0129 |
|  | GAM | Mixed | 0.321429 | 0.630102 | 0.357143 | 0.646684 | -0.0357 | -0.0166 |
|  | GAM | Invaded | 0.214286 | 0.616071 | 0.357143 | 0.664541 | -0.1429 | -0.0485 |
|  | GAM | Native | 0.289474 | 0.655125 | 0.285714 | 0.619962 | 0.0038 | 0.0352 |
|  | Ensemble | Mixed | 0.000000 | 0.610332 | 0.161607 | 0.638122 | -0.1616 | -0.0278 |
| Marsh frog | Ensemble | Invaded | 0.142857 | 0.603316 | 0.178571 | 0.654337 | -0.0357 | -0.0510 |
|  | Ensemble | Native | 0.131579 | 0.619114 | 0.183929 | 0.652168 | -0.0523 | -0.0331 |
|  | RF | Both | 0.285714 | 0.605867 | 0.250000 | 0.563170 | 0.0357 | 0.0427 |
|  | RF | Invaded | 0.250000 | 0.630740 | 0.357143 | 0.649904 | -0.1071 | -0.0192 |
|  | RF | Native | 0.250000 | 0.587372 | 0.244643 | 0.585906 | 0.0054 | 0.0015 |
|  | GLM | Both | 0.250000 | 0.591837 | 0.340179 | 0.639158 | -0.0902 | -0.0473 |
|  | GLM | Invaded | 0.250000 | 0.580357 | 0.340179 | 0.648023 | -0.0902 | -0.0677 |
|  | GLM | Native | 0.107143 | 0.475765 | 0.250000 | 0.580804 | -0.1429 | -0.1050 |
|  | BRT | Mixed | 0.3214286 | 0.5880102 | null | null | null | null |
|  | BRT | Natural | 0.2857143 | 0.6135204 | null | null | null | null |
|  | BRT | Anthropic | 0.1428571 | 0.5318878 | null | null | null | null |
|  | MaxEnt | Mixed | 0.250000 | 0.437500 | 0.750000 | 0.859375 | -0.5000 | -0.4219 |
|  | MaxEnt | Invaded | 0.250000 | 0.531250 | 0.750000 | 0.859375 | -0.5000 | -0.3281 |
|  | MaxEnt | Native | 0.000000 | 0.031250 | 0.750000 | 0.890625 | -0.7500 | -0.8594 |
|  | GAM | Mixed | 0.500000 | 0.687500 | 0.750000 | 0.891016 | -0.2500 | -0.2035 |
|  | GAM | Invaded | 0.500000 | 0.734375 | 0.750000 | 0.859375 | -0.2500 | -0.1250 |
|  | GAM | Native | 0.250000 | 0.468750 | 0.750000 | 0.883203 | -0.5000 | -0.4145 |
|  | Ensemble | Mixed | 0.000000 | 0.500000 | 0.125000 | 0.781250 | -0.1250 | -0.2813 |
| Pumpkinseed | Ensemble | Invaded | 0.125000 | 0.601563 | 0.375000 | 0.859375 | -0.2500 | -0.2578 |
|  | Ensemble | Native | 0.125000 | 0.453125 | 0.375000 | 0.809570 | -0.2500 | -0.3564 |
|  | RF | Both | 0.250000 | 0.593750 | 0.500000 | 0.718750 | -0.2500 | -0.1250 |
|  | RF | Invaded | 0.375000 | 0.687500 | 0.750000 | 0.844141 | -0.3750 | -0.1566 |
|  | RF | Native | 0.375000 | 0.656250 | 0.750000 | 0.828125 | -0.3750 | -0.1719 |
|  | GLM | Both | 0.125000 | 0.437500 | 0.750000 | 0.875000 | -0.6250 | -0.4375 |
|  | GLM | Invaded | 0.250000 | 0.609375 | 0.750000 | 0.875000 | -0.5000 | -0.2656 |
|  | GLM | Native | 0.000000 | 0.187500 | 0.625000 | 0.843750 | -0.6250 | -0.6563 |
|  | BRT | Mixed | 0.25 | 0.59375 | null | null | null | null |
|  | BRT | Natural | 0.375 | 0.609375 | null | null | null | null |
|  | BRT | Anthropic | 0.25 | 0.578125 | null | null | null | null |
|  | MaxEnt | Mixed | 0.224490 | 0.613911 | 0.244898 | 0.603759 | -0.0204 | 0.0102 |
|  | MaxEnt | Invaded | 0.306122 | 0.635985 | 0.285714 | 0.634465 | 0.0204 | 0.0015 |
|  | MaxEnt | Native | 0.183674 | 0.588713 | 0.277041 | 0.639681 | -0.0934 | -0.0510 |
|  | GAM | Mixed | 0.306123 | 0.625989 | 0.265306 | 0.622616 | 0.0408 | 0.0034 |
|  | GAM | Invaded | 0.326531 | 0.627655 | 0.285714 | 0.644200 | 0.0408 | -0.0165 |
|  | GAM | Native | 0.285714 | 0.625573 | 0.306122 | 0.653925 | -0.0204 | -0.0284 |
|  | Ensemble | Mixed | 0.061224 | 0.610995 | 0.142857 | 0.617576 | -0.0816 | -0.0066 |
| Zander | Ensemble | Invaded | 0.142857 | 0.623699 | 0.102041 | 0.626723 | 0.0408 | -0.0030 |
|  | Ensemble | Native | 0.142857 | 0.600791 | 0.142857 | 0.637573 | 0.0000 | -0.0368 |
|  | RF | Both | 0.224489 | 0.591420 | 0.183674 | 0.537714 | 0.0408 | 0.0537 |
|  | RF | Invaded | 0.204082 | 0.616826 | 0.183674 | 0.540608 | 0.0204 | 0.0762 |
|  | RF | Native | 0.204082 | 0.580591 | 0.204082 | 0.595190 | 0.0000 | -0.0146 |
|  | GLM | Both | 0.244898 | 0.600583 | 0.244898 | 0.600583 | 0.0000 | 0.0000 |
|  | GLM | Invaded | 0.244898 | 0.612245 | 0.244898 | 0.612245 | 0.0000 | 0.0000 |
|  | GLM | Native | 0.306122 | 0.629738 | 0.306122 | 0.629738 | 0.0000 | 0.0000 |
|  | BRT | Mixed | 0.2040816 | 0.5830904 | null | null | null | null |
|  | BRT | Natural | 0.1836735 | 0.607247 | null | null | null | null |
|  | BRT | Anthropic | 0.1428571 | 0.5501874 | null | null | null | null |
|  | MaxEnt | Both | 0.750000 | 0.937500 | 1 | 1 | -0.0625 | -0.2500 |
|  | MaxEnt | Invaded | 1.000000 | 1.000000 | 1 | 1 | 0.0000 | 0.0000 |
|  | MaxEnt | Native | 0.250000 | 0.375000 | 1 | 1 | -0.6250 | -0.7500 |
|  | GAM | Both | 0.500000 | 0.812500 | 1 | 1 | -0.1875 | -0.5000 |
|  | GAM | Invaded | 0.750000 | 0.937500 | 0.75 | 0.9375 | 0.0000 | 0.0000 |
|  | GAM | Native | 0.500000 | 0.652500 | 1 | 1 | -0.3475 | -0.5000 |
|  | Ensemble | Both | 0.000000 | 0.875000 | 0.75 | 1 | -0.1250 | -0.7500 |
| Killer shrimp | Ensemble | Invaded | 0.250000 | 0.937500 | 0.5 | 1 | -0.0625 | -0.2500 |
|  | Ensemble | Native | 0.250000 | 0.500000 | 0.5 | 1 | -0.5000 | -0.2500 |
|  | RF | Both | 0.250000 | 0.437500 | 0.750000 | 0.875000 | -0.5000 | -0.4375 |
|  | RF | Invaded | 0.250000 | 0.375000 | 0.500000 | 0.750000 | -0.2500 | -0.3750 |
|  | RF | Native | 0.250000 | 0.375000 | 0.750000 | 0.750000 | -0.5000 | -0.3750 |
|  | GLM | Both | 0.000000 | 0.312500 | 0.000000 | 0.312500 | 0.0000 | 0.0000 |
|  | GLM | Invaded | 0.250000 | 0.562500 | 0.250000 | 0.562500 | 0.0000 | 0.0000 |
|  | GLM | Native | 0.000000 | 0.312500 | 0.250000 | 0.651250 | -0.2500 | -0.3388 |
|  | BRT | Mixed | 0.25 | 0.5625 | null | null | null | null |
|  | BRT | Natural | 0.25 | 0.5 | null | null | null | null |
|  | BRT | Anthropic | 0.5 | 0.6875 | null | null | null | null |
|  | MaxEnt | Mixed | 0.176112 | 0.595139 | 0.118983 | 0.552609 | 0.0571 | 0.0425 |
|  | MaxEnt | Invaded | 0.152372 | 0.581074 | 0.127288 | 0.558513 | 0.0251 | 0.0226 |
|  | MaxEnt | Native | 0.135593 | 0.563298 | 0.233385 | 0.598082 | -0.0978 | -0.0348 |
|  | GAM | Mixed | 0.192706 | 0.599529 | 0.131271 | 0.554455 | 0.0614 | 0.0451 |
|  | GAM | Invaded | 0.135158 | 0.576099 | 0.123983 | 0.559091 | 0.0112 | 0.0170 |
|  | GAM | Native | 0.125424 | 0.563677 | 0.261180 | 0.642005 | -0.1358 | -0.0783 |
|  | Ensemble | Mixed | 0.096088 | 0.598502 | 0.054676 | 0.559908 | 0.0414 | 0.0386 |
| Signal crayfish | Ensemble | Invaded | 0.078852 | 0.577760 | 0.026114 | 0.555054 | 0.0527 | 0.0227 |
|  | Ensemble | Native | 0.074784 | 0.569222 | 0.054814 | 0.539206 | 0.0200 | 0.0300 |
|  | RF | Both | 0.169492 | 0.596880 | 0.122034 | 0.555010 | 0.0475 | 0.0419 |
|  | RF | Invaded | 0.142373 | 0.574685 | 0.108475 | 0.541913 | 0.0339 | 0.0328 |
|  | RF | Native | 0.037288 | 0.503034 | 0.108475 | 0.554936 | -0.0712 | -0.0519 |
|  | GLM | Both | 0.135593 | 0.564539 | 0.135593 | 0.564539 | 0.0000 | 0.0000 |
|  | GLM | Invaded | 0.094915 | 0.551738 | 0.094915 | 0.551738 | 0.0000 | 0.0000 |
|  | GLM | Native | 0.037288 | 0.476794 | 0.105085 | 0.548584 | -0.0678 | -0.0718 |
|  | BRT | Mixed | 0.1898305 | 0.6028383 | null | null | null | null |
|  | BRT | Natural | 0.1559322 | 0.5868773 | null | null | null | null |
|  | BRT | Anthropic | 0.1288136 | 0.5772939 | null | null | null | null |
|  | MaxEnt | Mixed | 0.250000 | 0.562500 | 0.500000 | 0.734375 | -0.2500 | -0.1719 |
|  | MaxEnt | Invaded | 0.250000 | 0.531250 | 0.500000 | 0.656250 | -0.2500 | -0.1250 |
|  | MaxEnt | Native | 0.500000 | 0.765625 | 0.500000 | 0.687500 | 0.0000 | 0.0781 |
|  | GAM | Mixed | 0.375000 | 0.500000 | 0.500000 | 0.671875 | -0.1250 | -0.1719 |
|  | GAM | Invaded | 0.375000 | 0.578125 | 0.375000 | 0.609375 | 0.0000 | -0.0313 |
|  | GAM | Native | 0.500000 | 0.718750 | 0.500000 | 0.703125 | 0.0000 | 0.0156 |
|  | Ensemble | Mixed | 0.125000 | 0.640625 | 0.375000 | 0.734766 | -0.2500 | -0.0941 |
| Sunbleak | Ensemble | Invaded | 0.125000 | 0.632813 | 0.250000 | 0.703125 | -0.1250 | -0.0703 |
|  | Ensemble | Native | 0.375000 | 0.734375 | 0.125000 | 0.719531 | 0.2500 | 0.0148 |
|  | RF | Both | 0.375000 | 0.640625 | 0.500000 | 0.671875 | -0.1250 | -0.0313 |
|  | RF | Invaded | 0.375000 | 0.656250 | 0.625000 | 0.750000 | -0.2500 | -0.0938 |
|  | RF | Native | 0.375000 | 0.640625 | 0.375000 | 0.734375 | 0.0000 | -0.0938 |
|  | GLM | Both | 0.375000 | 0.593750 | 0.500000 | 0.703125 | -0.1250 | -0.1094 |
|  | GLM | Invaded | 0.375000 | 0.609375 | 0.500000 | 0.671875 | -0.1250 | -0.0625 |
|  | GLM | Native | 0.250000 | 0.531250 | 0.250000 | 0.531250 | 0.0000 | 0.0000 |
|  | BRT | Mixed | 0.375 | 0.5625 | null | null | null | null |
|  | BRT | Natural | 0.25 | 0.59375 | null | null | null | null |
|  | BRT | Anthropic |  | 0.453125 | null | null | null | null |
|  | MaxEnt | Mixed | 0.250000 | 0.600000 | 0.225000 | 0.572531 | 0.0250 | 0.0275 |
|  | MaxEnt | Invaded | 0.275000 | 0.610000 | 0.250000 | 0.585016 | 0.0250 | 0.0250 |
|  | MaxEnt | Native | 0.225000 | 0.543125 | 0.213125 | 0.578875 | 0.0119 | -0.0358 |
|  | GAM | Mixed | 0.250000 | 0.579375 | 0.250000 | 0.603125 | 0.0000 | -0.0238 |
|  | GAM | Invaded | 0.250000 | 0.586250 | 0.250000 | 0.589391 | 0.0000 | -0.0031 |
|  | GAM | Native | 0.175000 | 0.550000 | 0.225000 | 0.578219 | -0.0500 | -0.0282 |
|  | Ensemble | Mixed | 0.000000 | 0.569375 | 0.150000 | 0.615789 | -0.1500 | -0.0464 |
| Wels catfish | Ensemble | Invaded | 0.225000 | 0.589375 | 0.150000 | 0.604555 | 0.0750 | -0.0152 |
|  | Ensemble | Native | 0.050000 | 0.562500 | 0.075000 | 0.570086 | -0.0250 | -0.0076 |
|  | RF | Both | 0.150000 | 0.550625 | 0.350000 | 0.647531 | -0.2000 | -0.0969 |
|  | RF | Invaded | 0.175000 | 0.558750 | 0.300000 | 0.636906 | -0.1250 | -0.0782 |
|  | RF | Native | 0.175000 | 0.510000 | 0.251250 | 0.574438 | -0.0763 | -0.0644 |
|  | GLM | Both | 0.200000 | 0.570625 | 0.250000 | 0.598766 | -0.0500 | -0.0281 |
|  | GLM | Invaded | 0.175000 | 0.579375 | 0.225000 | 0.576891 | -0.0500 | 0.0025 |
|  | GLM | Native | 0.150000 | 0.506250 | 0.213125 | 0.563156 | -0.0631 | -0.0569 |
|  | BRT | Mixed | 0.175 | 0.553125 | null | null | null | null |
|  | BRT | Natural | 0.175 | 0.543125 | null | null | null | null |
|  | BRT | Anthropic | 0.175 | 0.53625 | null | null | null | null |
